# [^18^F]PSMA-1007 PET is comparable to [^99m^Tc]Tc-DMSA SPECT for renal cortical imaging

**DOI:** 10.1186/s41824-023-00185-2

**Published:** 2023-11-24

**Authors:** Kristian Valind, David Minarik, Sabine Garpered, Eva Persson, Jonas Jögi, Elin Trägårdh

**Affiliations:** 1 Department of Translational Medicine, Lund University, Malmö, Sweden; 2 Wallenberg Centre for Molecular Medicine, Lund University, Lund, Sweden; 3 Department of Medical Imaging and Physiology, Skåne University Hospital, Lund, Sweden; 4 Radiation Physics, Skåne University Hospital, Malmö, Sweden; 5 Department of Medical Imaging and Physiology, Skåne University Hospital, Malmö, Sweden

**Keywords:** PSMA, DMSA, PET, SPECT, Renal function, Renal cortex

## Abstract

**Background:**

Scintigraphy using technetium-99m labelled dimercaptosuccinic acid ([^99m^Tc]Tc-DMSA), taken up in the proximal tubules, is the standard in functional imaging of the renal cortex. Recent guidelines recommend performing [^99m^Tc]Tc-DMSA scintigraphy with single photon emission computed tomography (SPECT). Prostate-specific membrane antigen (PSMA) targeted positron emission tomography (PET) is used for staging and localization of recurrence in prostate cancer. A high renal uptake is often seen on PSMA PET, concordant with known PSMA expression in proximal tubules. This suggests PSMA PET could be used analogous to [^99m^Tc]Tc-DMSA scintigraphy for renal cortical imaging. [^18^F]PSMA-1007 is a promising radiopharmaceutical for this purpose due to low urinary clearance. In this study, we aimed to compare [^18^F]PSMA-1007 PET to [^99m^Tc]Tc-DMSA SPECT regarding split renal uptake and presence of renal uptake defects, in patients with prostate cancer. Three readers interpreted PET and SPECT images regarding presence of renal uptake defects, with each kidney split into cranial, mid and caudal segments. Kidneys were segmented in PET and SPECT images, and left renal uptake as a percentage of total renal uptake was measured.

**Results:**

Twenty patients with prostate cancer were included. 2 participants had single kidneys; thus 38 kidneys were evaluated. A total of 29 defects were found on both [^99m^Tc]Tc-DMSA SPECT and [^18^F]PSMA-1007 PET. Cohen’s kappa for concordance regarding presence of any defect was 0.76 on a per-segment basis and 0.67 on a per-kidney basis. Spearman’s r for left renal uptake percentage between [^99m^Tc]Tc-DMSA SPECT and [^18^F]PSMA-1007 PET was 0.95.

**Conclusions:**

[^18^F]PSMA-1007 PET is comparable to [^99m^Tc]Tc-DMSA SPECT for detection of uptake defects in this setting. Measurements of split renal function made using [^18^F]PSMA-1007 PET are valid and strongly correlated to measurements made with [^99m^Tc]Tc-DMSA SPECT.

**Supplementary Information:**

The online version contains supplementary material available at 10.1186/s41824-023-00185-2.

## Background

Scintigraphy using technetium-99m labelled dimercaptosuccinic acid ([^99m^Tc]Tc-DMSA) is the standard in functional imaging of the renal cortex. Since the 1970s, [^99m^Tc]Tc-DMSA scintigraphy has seen use in detection of acute pyelonephritis and scarring after infection, in characterization of structural anomalies such as horseshoe kidney, and in determining each kidney’s relative percentage of the total renal function (Enlander et al. 1974; Mandell et al. 1997; Piepsz et al. 1999, 2009). The primary use of [^99m^Tc]Tc-DMSA scintigraphy is in pediatrics, with many patients being younger than 2 years of age. [^99m^Tc]Tc-DMSA is taken up in the proximal tubules (Willis et al. 1977), where it accumulates proportional to blood flow and functional cortical mass (Yee et al. 1981). Renal uptake of [^99m^Tc]Tc-DMSA correlates to other measurements of renal function such as renal plasma flow, serum creatinine, creatinine clearance and glomerular filtration rate (Kawamura et al. 1978; Groshar et al. 1991; Taylor et al. 1986). While planar imaging has long been the norm, single photon emission computed tomography (SPECT) may be more sensitive (Kim et al. 2019), result in better interobserver agreement (Einarsdottir et al. 2020), and be more suitable for quantification of left and right renal function percentages (Reichkendler et al. 2020). Thus, SPECT is recommended in the latest Society of Nuclear Medicine and Molecular Imaging (SNMMI) procedure standard/European Association of Nuclear Medicine (EANM) practice guideline (Vali et al. 2022).

Positron emission tomography (PET), often performed with computed tomography (CT) as hybrid PET/CT studies, plays an important role in staging and detection of recurrence in many malignancies. Prostate-specific membrane antigen (PSMA) has become the main target for PET in prostate cancer, where it is used for initial staging of high-risk prostate cancer and for detection of recurrence. PSMA, a transmembrane glycoprotein overexpressed in prostate cancer, is also expressed in nonmalignant tissues such as the renal proximal tubules (Silver et al. 1997; Kinoshita et al. 2006). Two PSMA targeted radiopharmaceuticals; [^68^Ga]Ga-PSMA-11 and [^18^F]PSMA-1007, both with considerable renal uptake (Afshar-Oromieh et al. 2016; Giesel et al. 2017), are being explored for imaging of renal cortical function analogous to [^99m^Tc]Tc-DMSA (Sarikaya et al. 2018; Valind et al. 2021; Ahmadi Bidakhvidi et al. 2022). The lower urinary clearance of [^18^F]PSMA-1007 compared to other common PSMA targeted radiopharmaceuticals (Giesel et al. 2017; Donswijk et al. 2022) may be advantageous for renal functional imaging. For both [^18^F]PSMA-1007 and [^68^Ga]Ga-PSMA-11, correlations between renal uptake and renal functional parameters such as estimated glomerular filtration rate (eGFR) have been demonstrated (Schierz et al. 2021; Valind et al. 2023). For [^68^Ga]Ga-PSMA-11, each kidney’s percentage of the total renal uptake has been shown to correlate to split renal function percentage as measured with technetium-99m labelled mercaptotriglycene ([^99m^Tc]Tc -MAG3) renography (Rosar et al. 2020). Sarikaya et al. initiated a study, approved in 2019, comparing renal uptake defects on [^99m^Tc]Tc-DMSA scintigraphy and [^68^Ga]Ga-PSMA-11 PET-CT in adult patients with pyelonephritis. Their study was interrupted due to the COVID-19 pandemic, with 2 case reports published separately (Sarikaya et al. 2021a, 2021b).

We have not however been able to find any direct comparison of DMSA SPECT and PSMA PET for more than one or two patients. Thus, the aim of this study was to compare [^99m^Tc]Tc-DMSA scintigraphy and [^18^F]PSMA-1007 PET regarding split renal uptake and regarding presence and extent of uptake defects.

## Methods

Twenty patients undergoing clinical [^18^F]PSMA-1007 PET/CT at the Department of Clinical Physiology and Nuclear Medicine, Skåne University Hospital, Malmö were included and underwent [^99m^Tc]Tc-DMSA scintigraphy within 2 weeks (median 7 days, range 2–11 days) of their PET-CT. The included patients were all male, with ages ranging from 61 to 83 years old (Table 1).

**Table 1:** Participant characteristics

Parameter	n
Participants	20
Kidneys	38

Parameter	Median (range)
Age (years)	69 (61–83)
Weight (kg)	86 (67–116)
Height (cm)	181 (164–194)
Serum creatinine (µmol/L)	92 (75–286)
Relative eGFR (ml/min/1.73m^2^)	77 (19–98)
Absolute eGFR (ml/min)	95 (23–131)

The PET/CT studies were performed on a GE Discovery MI (GE Healthcare, Milwaukee, WI, USA) according to clinical protocol, with image acquisition for 2 min/bed position starting 2 h post-injection of 4 MBq/kg of [^18^F]PSMA-1007 (range 286–460 MBq). PET images were reconstructed using Q.Clear (GE Healthcare, Milwaukee, WI, USA), a block-sequential regularisation expectation maximisation (BSREM) algorithm. A β-value of 800 was used for reconstruction (Trägårdh et al. 2020).

[^99m^Tc]Tc-DMSA scintigraphies were performed on a Siemens Symbia Intevo (Siemens Healthineers, Erlangen, Germany), after injection of [^99m^Tc]Tc-DMSA (median 123 MBq, range 116–127 MBq). SPECT/CT acquisition was performed for each patient, with an acquisition time of 20 min. SPECT data were reconstructed using ordered subset expectation maximization (OSEM), with 8 iterations and 9 subsets. Resolution recovery, scatter correction and attenuation correction was used, as well as a 7 mm gaussian post filter. In addition, planar imaging was performed according to clinical routine, with posterior, right posterior oblique (RPO) and left posterior oblique (LPO) acquisitions.

Each kidney was segmented as a separate volume of interest (VOI) in the PET and SPECT images using an in house-developed convolutional neural network (CNN), to reduce variability and time required for segmentation. Thorough manual review and correction of each VOI was performed. Segmentation examples are provided as Additional file 1.

Three nuclear medicine physicians, blinded to patient data and to each other’s readings, reviewed each PET, SPECT and planar acquisition without access to corresponding CT data. Images from all patients were reviewed in random order. Kidneys were divided into 3 segments (cranial pole, mid-kidney, and caudal pole). Each reader was asked to assess presence or absence of any uptake defects in each segment, as well as the total number of defects per kidney. Studies which the readers had classified differently were reinterpreted in consensus.

For each SPECT [^99m^Tc]Tc-DMSA dataset, the left renal uptake percentage (LRU%) was calculated as the number of counts in the left renal VOI divided by the total number of counts in the renal VOIs. For each [^18^F]PSMA-1007 PET dataset, left and right renal uptake (RU) was calculated by multiplying the mean standardized uptake value (SUVmean) of the left or right VOI by the volume size in milliliters. LRU% was then calculated by dividing the left RU by the sum of the left and right RU values.

Concordance between modalities regarding presence or absence of any uptake defect in each renal segment was tested using unweighted Cohen’s kappa, while weighted Cohen’s kappa was used to test concordance regarding the number of uptake defects found per kidney. Correlations between LRU% measurements from the different modalities were evaluated using Spearman rank correlation. In addition, Pearson’s correlation coefficient was calculated for PET LRU% against SPECT LRU% to allow comparison to other studies. Bland–Altman analysis (Altman and Bland 1983) was used to examine differences between LRU% measurements in PET and SPECT data.

## Results

**Fig. 1:**
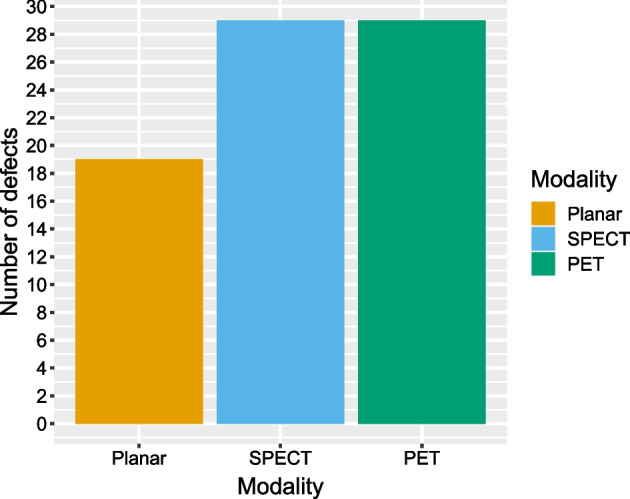
Total number of defects found per modality, final consensus interpretations. *SPECT* single photon emission computed tomography; *PET* positron emission tomography

Two participants had single kidneys. Thus 38 kidneys (114 renal segments) were evaluated. In both SPECT and PET images, a total of 29 defects were found (Fig. 1). Unweighted Cohen’s kappa for concordance between SPECT and PET regarding presence or absence of any defect was 0.76 on a per-segment basis (Table 2) and 0.67 on a per-kidney basis (Table 3). A similar weighted Cohen’s kappa of 0.66 found when comparing the number of defects found per kidney (Table 4). Concordance between planar and tomographic images was similar; Cohen’s kappa for presence or absence of any defect was 0.71 against SPECT as well as against PET on a per-segment basis, along with 0.89 against SPECT and 0.78 against PET on a per-kidney basis.

**Table 2:** Any defect, per segment

Any defect in segment (SPECT)	Any defect in segment (PET)	
	No	Yes
No	78	5
Yes	5	23
Cohen's kappa: 0.76 (95% CI: 0.62 – 0.90)		

Total number of segments: 111. 1 kidney was not visualized by any reader

**Table 3:** Any defect, per kidney

Any defect in kidney (SPECT)	Any defect in kidney (PET)	
	No	Yes
No	18	4
Yes	2	13
Cohen's kappa: 0.67 (95% CI: 0.43 – 0.91)		

Total number of kidneys: 37. 1 kidney was not visualized by any reader

**Table 4:** Number of defects, per kidney

Number of defects (SPECT)	Number of defects (PET)			
	0	1	2	3
0	18	3	1	0
1	2	3	1	0
2	0	1	4	1
3	0	1	0	1
5	0	0	0	1
Cohen's kappa (weighted): 0.66 (95% CI: 0.49 – 0.84)				

Total number of kidneys: 37. 1 kidney was not visualized by any reader

**Fig. 2:**
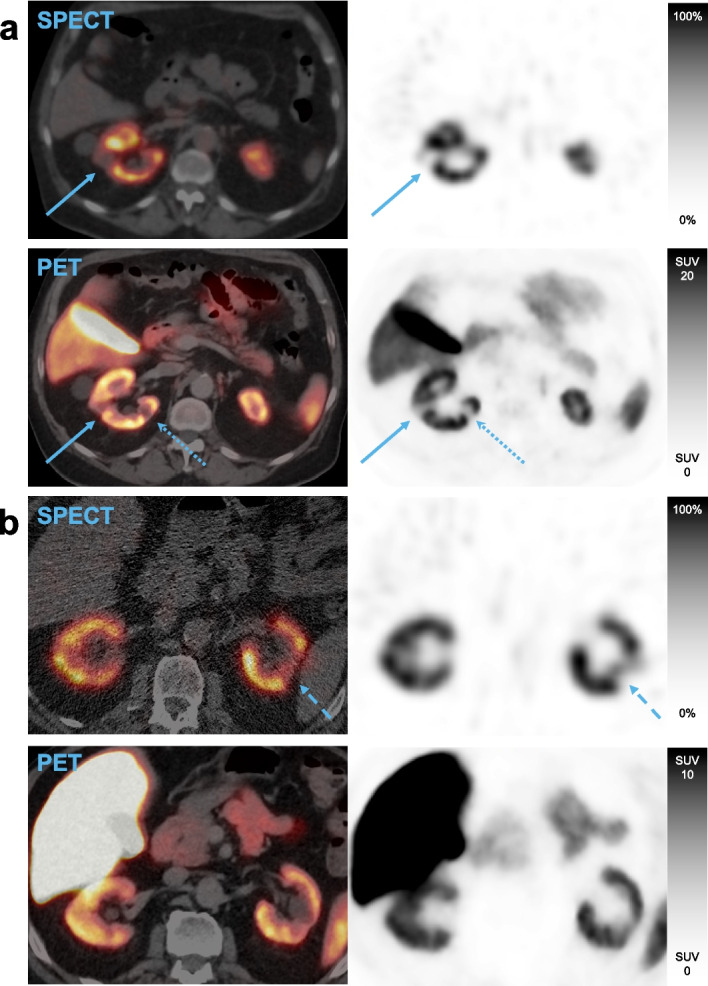
Example PET and SPECT images from two patients. Solid arrows in **a** indicate a defect found by readers on both PET and SPECT. Dotted arrows in **a** indicate a defect found on PET but not on SPECT. Dashed arrows in **b** indicate a defect readers found on SPECT but not on PET images. *SPECT*: single photon emission computed tomography; *PET*: positron emission tomography

Representative images of concordant and discordant findings are provided in Fig. 2. Most defects were identified as cysts using CT data acquired with the PET and SPECT images, with uptake defects in 3 participants representing renal scarring. In total, 7 defects were identified in SPECT images that were not found in PET images. Correlating to CT data, 5 of these defects were found to represent cysts of around 1 cm^3^, with no explanation found for the remaining 2. Conversely, 7 defects were found in SPECT but not in PET. Local thinning of the parenchyma corresponded to 3 of these, one represented a 1.5 cm^3^ cyst, one a 2 cm^3^ parapelvic cyst, and for one defect no explanation was found. In the remaining case two adjacent cysts were interpreted as one defect on PET but 2 defects on SPECT. Result tables where cysts have been excluded are provided as Additional file 2.

In one participant, none of the readers identified the right kidney regardless of modality. CT revealed a small polycystic kidney, with a low uptake of [^99m^Tc]Tc-DMSA and [^18^F]PSMA-1007.

**Fig. 3:**
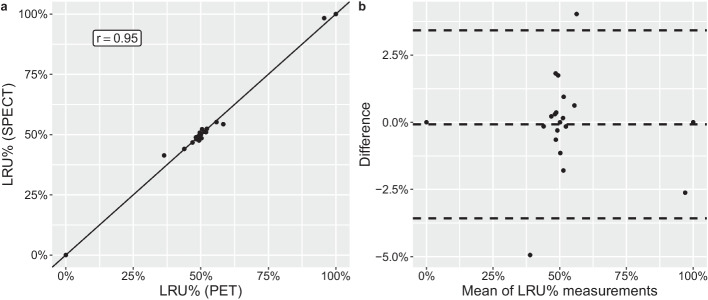
Correlation (**a**) and Bland–Altman (**b**) plots of left renal uptake percentage (LRU%) measured with SPECT and PET. Dashed lines indicate mean difference and 95% limits of agreement (mean ± 1.96 SD). *SPECT*: single photon emission computed tomography; *PET*: positron emission tomography; *LRU%*, left renal uptake percentage; *SD*, standard deviation

Spearman’s r between SPECT and PET LRU% was 0.95 (Fig. 3). Pearson’s r was 0.99 for the same comparison. Excluding the two participants with single kidneys resulted in a Spearman’s r of 0.93. Weaker correlations were found when comparing planar to tomographic data (Spearman’s r 0.80 against SPECT and 0.79 against PET).

## Discussion

In this study, we performed a direct comparison of [^99m^Tc]Tc-DMSA SPECT and [^18^F]PSMA-1007 PET for detection of renal uptake defects and measurement of split renal function. We found substantial agreement regarding presence and number of defects (Cohen’s kappa = 0.66), and an excellent correlation between LRU% measurements between the different modalities (Spearman’s r = 0.95). Differences in spatial resolution could to a large degree explain differences in defect findings between modalities. Defects found in PET but not SPECT mainly represented small cysts, while almost half of the defects found in SPECT but not PET represented cortical thinning and could be seen as false positives. A higher spatial resolution could thus be beneficial regardless of the size of the lesion in question.

These results show that [^18^F]PSMA-1007 PET is a valid method for measurement of split renal function and that it can be used to detect localized functional defects, such as scars. Results comparable to those of a 20 min [^99m^Tc]Tc-DMSA SPECT can thus be obtained with [^18^F]PSMA-1007 PET in around 4 min, assuming the kidneys fit in 2 bed positions. Importantly, a low uptake in one kidney on a [^18^F]PSMA-1007 PET, as performed in a prostate cancer setting, indicate reduced function and may warrant further investigation. Furthermore, our results serve as an initial proof of concept for the use of [^18^F]PSMA-1007 PET as a high resolution method for imaging localized renal damage or impairment.

The concept of using PSMA PET for detection of renal cortical defects was pioneered by Sarikaya et al. in a series of publications, including the first published case report of such use, as well as cases from a prospective study comparing [^68^Ga]Ga-PSMA-11 PET to [^99m^Tc]Tc-DMSA SPECT (Sarikaya et al. 2018, 2021a, b; Sarikaya 2019; Sarikaya and Sarikaya 2019).

Correlations between PET parameters and measurements of total renal function have been established for both [^68^Ga]Ga-PSMA-11 (Schierz et al. 2021) and [^18^F]PSMA-1007 (Valind et al. 2023), strengthening the case for renal PSMA PET.

Rosar and coauthors compared [^99m^Tc]Tc -MAG3 scintigraphy and [^68^Ga]Ga-PSMA-11 PET for measurement of split renal function (Rosar et al. 2020). They found a strong correlation (Pearson’s r = 0.91) between these two modalities. [^99m^Tc]Tc-DMSA and [^18^F]PSMA-1007, used in our study, have significantly lower urinary clearance than [^99m^Tc]Tc -MAG3 and [^68^Ga]Ga-PSMA-11. This allows evaluation of the renal cortex for defects, in addition to measurement of split function. The slightly stronger correlation in our study (Pearsons’s r = 0.99) could in part be due to less activity in renal calyces than can be the case with [^68^Ga]Ga-PSMA-11 PET.

Ahmadi Bidakhvidi and coauthors published a case report where additional uptake defects were found with [^18^F]PSMA-1007 PET compared to [^99m^Tc]Tc-DMSA SPECT (Ahmadi Bidakhvidi et al. 2022). Comparable to the present study, differences in spatial resolution may be the primary explanation of this difference.

The main limitations of our study are the small number of the participants and the narrow demographics, which limit the generalizability of our results. For example, renal uptake of [^18^F]PSMA-1007 in children remains to be investigated. Since the primary use of [^99m^Tc]Tc-DMSA scintigraphy is in pediatrics, we consider this a major limitation. Additionally, most defects found corresponded to renal cysts rather than scars.

## Conclusions

Measurements of split renal function made using [^18^F]PSMA-1007 PET are valid and strongly correlated to measurements made with [^99m^Tc]Tc-DMSA SPECT, in patients with prostate cancer. In this setting, [^18^F]PSMA-1007 PET is comparable to [^99m^Tc]Tc-DMSA SPECT for detection of uptake defects. Our results represent an initial proof of concept of [^18^F]PSMA-1007 PET as a high resolution imaging method for localized renal damage or functional impairment. It remains to be investigated if these results are valid in a mixed sex pediatric population, where [^99m^Tc]Tc-DMSA scintigraphy plays a more important role.

### Supplementary Information

**Additional file 1.** Segmentation examples.

**Additional file 2.** Result tables with cysts excluded.

## Data Availability

Data and material are available from the corresponding author upon reasonable request.

## Abbreviations

BSREM: Block-sequential regularisation expectation maximisation
CI: Confidence interval
CNN: Convolutional neural network
CT: Computed tomography
DMSA: Dimercaptosuccinic acid
eGFR: Estimated glomerular filtration rate
LPO: Left posterior oblique
LRU%: Left renal uptake percentage
MAG3: Mercaptotriglycene
OSEM: Ordered subset expectation maximisation
PET: Positron emission tomography
PSMA: Prostate-specific membrane antigen
RPO: Right posterior oblique
RU: Renal uptake
SD: Standard deviation
SPECT: Single photon emission computed tomography
VOI: Volume of interest
