# Supplementary material for: [18F]PSMA-1007 PET is comparable to [99mTc]Tc-DMSA SPECT for renal cortical imaging
Source: Eur J Hybrid Imaging. 2023 Nov 24;7:25. doi: 10.1186/s41824-023-00185-2 (PMC10667166; doi:10.1186/s41824-023-00185-2)
Supplement: Supplementary file 1 — Additional file 1. Segmentation examples. [file 41824_2023_185_MOESM1_ESM.docx]

# Supplement 1: Segmentation examples

PET (a-d) and SPECT (e-h) example segmentations from a representative patient, maximum intensity projection (a, e), transversal (b, f), coronal (c, g), and sagittal (d, h) views. Crimson indicates the right kidney segmentation, and cyan the left kidney segmentation.
