# Supplementary material for: [18F]PSMA-1007 PET is comparable to [99mTc]Tc-DMSA SPECT for renal cortical imaging
Source: Eur J Hybrid Imaging. 2023 Nov 24;7:25. doi: 10.1186/s41824-023-00185-2 (PMC10667166; doi:10.1186/s41824-023-00185-2)
Supplement: Supplementary file 2 — Additional file 2. Result tables with cysts excluded. [file 41824_2023_185_MOESM2_ESM.docx]

# Supplement 2: Tables without cysts

1. Any defect, per segment.

| **Any defect in segment** **(SPECT)** | **Any defect in segment (PET)** | |
| --- | --- | --- |
|  | **No** | **Yes** |
| No | 103 | 2 |
| Yes | 3 | 3 |
| Cohen's kappa: 0.52 (95% CI: 0.15 - 0.89) | | |

Total number of segments: 111. 1 kidney was not visualized by any reader.

2. Any defect, per kidney.

| **Any defect in kidney  (SPECT)** | **Any defect in kidney (PET)** | |
| --- | --- | --- |
|  | **No** | **Yes** |
| No | 30 | 2 |
| Yes | 2 | 3 |
| Cohen's kappa: 0.54 (95% CI: 0.14 - 0.94) | | |

Total number of kidneys: 37. 1 kidney was not visualized by any reader.

3. Number of defects, per kidney.

| **Number of defects (SPECT)** | **Number of defects (PET)** | | |
| --- | --- | --- | --- |
|  | **0** | **1** | **2** |
| **0** | 30 | 1 | 1 |
| **1** | 2 | 2 | 0 |
| **2** | 0 | 1 | 0 |
| Cohen's kappa (weighted): 0.43 (95% CI: 0.08 - 0.79) | | | |

Total number of kidneys: 37. 1 kidney was not visualized by any reader.
